# Increasing Minimally Processed Food Intake in Depression via Commercial Meal Delivery: Qualitative Accounts of Participant Experiences

**DOI:** 10.3390/nu18121852

**Published:** 2026-06-09

**Authors:** Celina R. Furman, Elena L. Pokowitz, Sushmitha Peddireddy, Imogen Bylinsky, Jacki D. Zhang, Ingrid A. Worth, Kendrin R. Sonneville, Ashley N. Gearhardt

**Affiliations:** 1 Department of Psychology, University of Michigan, 530 Church Street, Ann Arbor, MI 48109, USA; 2 School of Public Health, University of Michigan, 1415 Washington Heights, Ann Arbor, MI 48109, USA

**Keywords:** depression, mental health, food quality, dietary intervention, qualitative analysis

## Abstract

**Background/Objectives:** Several randomized controlled trials have found that dietary interventions promoting self-guided shifts away from ultra-processed foods (UPFs) and toward minimally processed, nutrient-dense foods may alleviate depressive symptoms. However, translating these interventions into scalable and sustainable real-world approaches remains a key challenge. Adopting a minimally processed dietary pattern requires sustained effort (e.g., meal planning, shopping, and preparation) within environments where UPFs are pervasive and convenient. These demands may be especially burdensome for individuals experiencing depressive symptoms. Consequently, interventions that rely heavily on individual effort may be difficult to maintain. Commercial meal delivery services may offer a structural solution by reducing logistical and cognitive barriers to dietary change, yet little is known about how individuals with depressive symptoms experience this approach. **Methods:** In a parent study, we conducted a randomized pilot study of a meal delivery service designed to provide minimally processed meals to adults with moderate to moderately severe depressive symptoms; here we report qualitative findings from post-intervention interviews with participants assigned to the meal delivery condition (*n* = 20). **Results:** Participants appreciated reductions in preparatory effort and mental load, which supported adherence. Dietary changes were also linked to improved mood through increased energy, mood stability, and more positive self-evaluation. However, social settings were a near-universal barrier, and acceptability depended on palatability, compatibility with personal preferences, and perceived autonomy. Several participants also described a temporal adjustment process (early cravings/withdrawal followed by adaptation). **Conclusions:** Overall, these findings suggest participant-informed priorities for future testing and refinement of scalable meal delivery interventions for depression, including personalization and choice, quality control, and support for social and withdrawal-related challenges.

## 1. Introduction

Depression is a leading cause of disability worldwide, and identifying scalable, modifiable risk factors remains a public health priority [1]. Diet quality has emerged as one such factor, with accumulating evidence linking high consumption of ultra-processed foods to the onset and exacerbation of depressive symptoms [2,3,4]. Randomized controlled trials have also demonstrated that dietary interventions encouraging participant-led shifts from ultra-processed foods toward minimally processed, nutrient-dense foods can reduce depressive symptoms among adults with elevated depressive symptoms [5,6,7,8]. These findings have advanced the field and suggest that dietary change may serve as a complementary strategy for the prevention and treatment of depressive disorders. However, translating these interventions into scalable and sustainable real-world approaches remains a central challenge.

Adopting a minimally processed dietary pattern requires sustained behavior change, including meal planning, grocery shopping, food preparation, and ongoing decision-making. These demands can undermine long-term adherence [9,10], particularly in a food environment in which ultra-processed foods are ubiquitous, inexpensive, heavily marketed, and engineered for convenience. In the United States, ultra-processed foods account for nearly 60% of total daily energy intake and dominate the majority of packaged and prepared foods available across grocery and convenience stores, vending machines, fast food and restaurant menus, and app-based food delivery platforms [11]. As ultra-processed options function as the default across eating contexts, shifting toward minimally processed foods requires not only knowledge, but sustained effort to navigate environmental cues and structural convenience. For individuals experiencing depressive symptoms (e.g., fatigue, cognitive burden, avolition), sustained implementation may be especially challenging due to impairments in executive functioning and daily task completion [12]. Interventions that rely heavily on individual effort may therefore place the greatest demands on those with the fewest available psychological resources, limiting feasibility and long-term sustainability despite potential benefit.

Commercial meal-delivery services may present a novel structural solution. These services already operate at scale and have grown rapidly in popularity due to their convenience and reduction in planning and preparation burden [13,14]. By providing fully prepared meals, they reduce decision-making demands and logistical barriers such as grocery shopping, time required for meal preparation, and the physical effort involved in cooking. Importantly, these services are not inherently “healthy”; commercially available options span a range of nutritional quality, often including meals high in refined ingredients, added sugars, or sodium-rich sauces [14]. However, because they function as flexible platforms rather than fixed dietary prescriptions, they can be strategically leveraged to deliver meals composed primarily of minimally processed ingredients. In this way, meal delivery services may function as a practical scaffold that lowers the effort required to translate dietary intentions into action, which may be especially valuable during periods of elevated depressive symptoms characterized by diminished motivation and energy.

Despite their increasing availability, meal delivery services have not, to our knowledge, been intentionally leveraged as a strategy to facilitate minimally processed dietary change for depression. In addition, little is known about how individuals with depressive symptoms experience such an approach. To address this gap, we conducted a randomized pilot evaluation comparing self-directed nutritional guidance (i.e., nutritional guidance condition) with nutritional guidance plus a commercial meal delivery service intentionally structured to provide minimally processed meals as a strategy to reduce depressive symptoms among adults experiencing moderate to moderately severe symptoms (i.e., meal delivery condition); primary trial aims, protocols, and outcomes are reported by Furman et al. (2026) [15]. Quantitative findings provided preliminary insight into short-term outcomes, including self-reported dietary change and adherence, meal acceptability and similarity to participants’ usual diets, perceived impact on mental health and depressive symptoms, and safety regarding disordered eating-related outcomes [15,16]. However, such measures cannot fully capture how participants experienced and integrated pre-made, minimally processed meals into their everyday routines.

Accordingly, this paper presents qualitative findings from post-intervention debriefing interviews with participants in the meal-delivery condition, designed to provide an in-depth examination of their experiences with the intervention. Notably, because participants in the meal delivery condition also received nutritional guidance, findings should be interpreted as reflecting participants’ experiences with nutritional guidance facilitated by meal delivery, rather than meal delivery in isolation. The objectives of these analyses were to explore perceived benefits and barriers, clarify under what circumstances meal delivery services may be most supportive, and identify opportunities for refinement when challenges arise. We also sought to understand how participants interpreted and experienced food–mood associations during the intervention and more broadly. Together, these insights will inform actionable recommendations for improving meal delivery-supported dietary interventions for depression care.

## 2. Materials and Methods

### 2.1. Transparency and Openness

This study was approved by the University of Michigan Institutional Review Board (HUM00231527), and the parent trial was preregistered on ClinicalTrials.gov (NCT06242665). The present manuscript reports a qualitative follow-up analysis that complements the primary trial aims and outcomes reported by Furman et al. (2026; see also for the detailed intervention protocol) [15]. The qualitative analytic plan for all interviewed participants was preregistered on the Open Science Framework (OSF) prior to reviewing interview data (https://osf.io/ad3n4). The present manuscript specifically elaborates on qualitative data from participants in the meal-delivery condition, representing a subset of the preregistered qualitative analysis. We focus on this condition to allow sufficient depth in reporting and interpretation of participants’ experiences; qualitative findings from the nutritional guidance-only condition will be reported separately. To protect participant confidentiality, de-identified interview transcripts will be made available upon reasonable request and contingent on approval of a data use agreement and applicable institutional requirements.

### 2.2. Qualitative Sample Characteristics

Participants were recruited from UMHealthResearch (https://umhealthresearch.org) and social media platforms between 9 December 2023 and 28 July 2024 for a study examining how dietary change relates to mood. Interested individuals completed an online screening questionnaire in REDCap to assess eligibility and ability to complete study procedures. Key inclusion criteria were as follows: (a) age ≥ 18 years; (b) fluent in English; (c) ownership of an Android or iPhone smartphone with ability to access the phone frequently throughout the day; (d) willingness to complete three in-person study visits and follow dietary instructions; (e) elevated depressive symptoms at consent (Patient Health Questionnaire [PHQ-8] [17] ≥10); (f) evidence of current ultra-processed food intake (endorsement of at least two ultra-processed foods consumed the day prior to screening); (g) no recent restrictive eating disorder (past five years); (h) no frequent night shift or highly irregular work schedules; (i) no severe allergies or dietary restrictions that could not be accommodated; and (j) elevated picky eating (Adult Picky Eating Questionnaire [18] score ≤ 3.4).

After eligibility was confirmed during a consent call and informed consent was obtained, participants were randomized to either the nutritional guidance or the meal delivery condition. The meal delivery sample had a mean age of 38.80 years (*SD* = 12.43); predominantly identified as female (80.0%), White (90.0%), and highly educated (75.0% has a bachelor’s or higher); and, on average, had moderate to moderately severe depressive symptoms at Visit 1 (assessed via PHQ-8; *M* = 14.45, *SD* = 2.93). Participants varied in concurrent mental health treatment, including psychotherapy, medication, both, or neither, and the study did not require changes to usual mental health care. Full inclusion/exclusion criteria and participant demographic and clinical characteristics are included in the primary aims paper [15].

### 2.3. Intervention Description

The dietary intervention period lasted two weeks and occurred between Visit 2 (pre-intervention) and Visit 3 (post-intervention). At the end of Visit 2, all participants were instructed to consume a diet consisting primarily of minimally processed foods beginning the next day. Participants received a handout containing nutritional guidance describing recommended foods and beverages to consume and to avoid (Supplementary Materials Table S1), along with a sample menu illustrating how to structure meals and snacks in accordance with a minimally processed dietary pattern.

To facilitate following of the nutritional guidance, participants received three meals per day (breakfast, lunch, and dinner) delivered to their home address for a total of 21 meals per week for two weeks. Meals were selected by the project coordinator through a commercial meal delivery service website from its Mediterranean, Paleolithic, or Whole30 categories, as these dietary patterns generally emphasize minimally processed ingredients and limit added sugars and refined carbohydrates [19,20,21]. Meal selection was further informed by allergies and food preferences collected during the consent call through a questionnaire provided by the meal delivery company. The questionnaire assessed (1) whether participants had any allergies to egg, sesame, shellfish, tree nuts, soy, or wheat and (2) preferences for ingredients that could be found in delivered foods, assessed on a 6-point Likert scale ranging from 1 (“Like Extremely”) to 6 (“Dislike Extremely”); the full list of ingredients assessed is included in Supplementary Materials). Meals containing ingredients participants disliked (i.e., rated > 3 on the Likert scale) or reported allergies to were not selected.

Participants were recommended to eat three meals and at least one snack per day but were explicitly instructed to prioritize their typical eating pattern if it differed (e.g., fewer meals and more snacks). If preparing a meal was not feasible or additional food was needed to manage hunger, participants were instructed to eat ad libitum from foods consistent with the nutritional guidance. To minimize withdrawal effects unrelated to dietary change, participants were instructed to maintain their usual caffeine and alcohol intake. Individualized guidance was provided to modify beverages in line with the nutritional recommendations (e.g., removing added sweeteners or changing mixers).

Co-author KS, a registered dietitian on the study team, contributed to vendor selection, development of meal selection criteria, and creation of supplemental nutritional guidance materials. The general nutritional composition of meals was confirmed with a company representative during the vendor selection process. A full description of the nutritional guidance, intervention procedures, and personalized meal selections is provided on the OSF project page (https://osf.io/pcufr/) and in the Supplementary Materials accompanying the primary aims paper [15].

### 2.4. Debriefing Interview Procedures

At the end of Visit 3, participants completed an in-person, fully structured debriefing interview about their experiences during the two-week intervention (see Supplementary Materials for the full interview guide). Interviews were conducted verbally by a trained student research assistant or other lab staff member, audio-recorded using a digital recorder, and uploaded to a secure lab server. In addition, interviewers documented detailed field notes in REDCap (captured as close to verbatim as possible) during interviews as a backup in cases of recording failure (*n* = 1) or participant non-consent (*n* = 2). Audio files were professionally transcribed by Scribie and reviewed by the research team for accuracy prior to analysis.

The interview guide included questions about participant experiences implementing the nutritional guidance and using the meal delivery service (e.g., overall experience, best aspects, and biggest obstacles), evaluation of the meals, perceived similarity to a typical diet, perceived impact on mental health, and intentions to continue after the study. Participants were also asked about cravings and withdrawal-like experiences and whether these affected mood or adherence, as well as whether other study procedures (e.g., ecological momentary assessment [EMA], Fitbit use) influenced mood or adherence. Finally, interviews probed participants’ broader perceptions of food–mood associations.

### 2.5. Thematic Analysis

A thematic analysis was conducted on transcriptions of the audio interview recordings to gain insight into the feasibility of the intervention (nutritional guidance + meal delivery service) in addition to the general experiences of participants in the study. Audio recordings from 18 participants were transcribed (one recording had an audio failure halfway through). Two participants did not consent to recording their interviews, so the research team used notes taken by the Visit 3 research assistant for coding purposes.

NVivo 15 qualitative analysis software was used to analyze the transcriptions in a hybrid deductive-inductive approach to coding within a reflexive thematic framework [22]. A deductive approach was used for two primary, pre-defined themes: (1) intervention feasibility and acceptability, and (2) intervention convenience and accessibility. An inductive approach was used to identify additional exploratory themes that emerged from the data.

The research team developed the codebook through a hybrid human/artificial intelligence (AI) approach using the NVivo AI Assistant. Primary coders (EP, IB, SP) initially created parent codes to be applied to the transcriptions. NVivo’s AI Assistant was then used to suggest child codes and definitions, which were reviewed and altered by primary coders to best fit the data. After training on the codebook, all transcripts and note page(s) were then re-coded and reviewed by all three primary coders to come to a group consensus. A final, validated codebook was created through this consensus discussion process (see Supplementary Materials). Codes were grouped into deductive themes and inductive subthemes by both primary (EP, IB, SP) and secondary coders (CF). Finally, secondary coder CF produced an integrative narrative account that emphasized relationships among themes, key tensions, and temporal patterning.

Primary and secondary coders did not conduct any of the debriefing interviews. Positionality statements are included in the Supplementary Materials.

## 3. Results

Analyses initially revealed multiple inductive themes related to our primary, deductive categories: (1) intervention feasibility and acceptability (nine inductive themes) and (2) intervention convenience and accessibility (three inductive themes). However, review of the qualitative data showed that these two deductive categories were highly interrelated in participants’ accounts. Convenience and accessibility consistently shaped how feasible and acceptable the intervention felt, making it difficult to interpret these domains as distinct or independent. Instead, they functioned together to influence participants’ engagement with and ability to adhere to the nutritional guidance and meal delivery program. To better reflect this integration, we organized findings into six broader thematic domains, each comprising related subthemes, which more accurately represent the interconnected processes through which participants engaged with the provided nutritional guidance and the supports in this study (a mapping of initial themes to the final domains is included in Supplementary Materials).

Additionally, although interviews probed nutritional guidance and meal delivery separately, participants typically described them as a single, integrated experience: the meals made minimally processed eating concrete and readily available, while the nutritional guidance provided rules and options when replacing or supplementing delivered meals. Across the initial themes, a central tension shaped engagement: participants valued the convenience and structure of the meal delivery program that reduced the daily mental load and effort associated with mealtimes, yet acceptability depended on palatability, alignment with personal preferences, and autonomy. When meals felt appetizing and choice-concordant, convenience translated into adherence and perceived benefits; when meals felt unpalatable, repetitive, or mismatched, the same structure could feel restrictive, undermine adherence, and heighten negative emotions. These integrations and experienced tensions provided our team with vital information as we organized our initial themes into the final six domains that narratively describe participant experiences in the current study.

The six thematic domains that follow characterize how (1) the intervention supported adherence by reducing preparatory-related burden, (2) learning and awareness developed through the intervention, (3) palatability and perceived autonomy shaped acceptability, (4) social contexts challenged adherence, (5) cravings and withdrawal-like experiences evolved over time, and (6) participants understood and experienced food–mood connections.

### 3.1. Domain 1: Structural Scaffolding Reduced Preparatory Burdens (Table 1)

Participants consistently framed the meal delivery service as a practical scaffold that made adherence easier by reducing effort, time, and decision-making. Participants emphasized *convenience*, noting that the provided meals reduced the need for meal planning, grocery shopping, and preparation/cooking while making mealtime routines faster and simpler. This, in turn, *reduced mental load*, with many describing less decision fatigue and food-related rumination, making eating feel “less stressful or strenuous” (P047). Some participants additionally noted that the meals allowed them to reclaim time and focus on other interests and responsibilities, such as spending time with pets or going to the gym. Participants highly valued *reliable access to healthy foods*, including reassurance from having prepared meals consistently available at home and, for some, being able to “trust” that what they were eating was healthy (P033, P037); a subset additionally noted that having meals provided at no cost reduced financial burden during the two-week period and improved nutritional security. Several participants further described *structured intake*, reporting that having “ready-to-eat, healthy meals” made previously sporadic eating patterns more regular and helped them eat higher-quality foods more consistently (P012). At the same time, several participants identified limitations, including *mismatch with lifestyle preferences* when they preferred greater autonomy or freshness (e.g., missing cooking, disliking reheated meals) and *implementation friction*, which was described as both a practical and values-based drawback: isolated delivery issues, meals being “really difficult to open,” and the “amount of plastic” and associated waste “bummed” a few of the participants out (P022, P012, P047).

**Table 1:** Subthemes and illustrative quotations related to Domain 1.

Subthemes	Illustrative Quotations
Increased convenience	*“It just made my life so much easier to have ready-to-go healthy meals. I struggle with having enough time to deal with all the decision-making and shopping and preparing of proper meals, so this was a huge game-changer for me.”* (P012) *“Most of it was food that I don’t normally eat because I don’t have the time really to prepare it, like the fish and stuff. So that was great to be able to have food that I enjoyed and not just food that’s convenient.”* (P022)
Reduced mental load	*“It was a huge relief of not having to worry about what to plan for meals and what to do for grocery shopping…I felt like I was on a little mini vacation for two weeks.”* (P044) *“[It’s] giving me a good attitude because I don’t have to come home and cook and I can spend the time with my dogs. I only fixed their meals and it’s just freed up a lot of my time.”* (P003)
Reliable access to healthy foods	*“When I don’t have to think about what I want to eat when I get hungry, and I know that there’s food already prepped for me, it kind of gives me comfort.”* (P033)
Structured intake	*“My regular diet had been very not really structured, and when I did remember to eat, there were foods that were either highly processed or not foods that made me feel very good…even during the study, my eating schedule was still pretty chaotic, but I had those meals so, I was actually eating more frequently and eating more vegetables and proteins.”* (P034)
Mismatch with lifestyle preferences	*“Something more like HelloFresh… so it would be fresh, freshly cooked… I’m not really much of a leftovers person, so heating up pre-cooked food didn’t do it for me.”* (P031)
Implementation friction	*“The packaging was really difficult to open… I pretty much always had to bring scissors with me to cut [it], and that was difficult.”* (P022)

Note. Subthemes were derived from qualitative analysis of participant interviews. Illustrative quotes are provided as representative examples and have been lightly edited for readability while preserving participant meaning. Ellipses indicate omitted text, and bracketed text indicates clarifying information added by the authors.

### 3.2. Domain 2: Nutritional Guidance, Increased Dietary Awareness, and Skill-Building (Table 2)

Participants described several learning processes in response to the intervention and study-provided materials. Many used the nutritional guidance handouts as an *in-the-moment reference tool* to “look back on,” which reduced uncertainty and simplified choices through clear “eat/avoid” rules (P001, P033). These materials supported skill-building through *substitution and planning strategies* derived from the nutritional guidance (e.g., swapping fruit for sweets, flavored seltzer for soda, and bringing compliant foods or snacks into higher-risk social settings). For example, two participants described a clear shift toward fruit as a preferred substitute for sweet foods, one noting that she “fell in love with apples” (P016). The delivered meals further supported learning by serving as practical *“how-to” models* of minimally processed meals that participants felt they could recreate when cooking on their own. More broadly, participation also *heightened awareness of dietary patterns*, including greater recognition of baseline reliance on ultra-processed foods due to habit, convenience, and mood. Finally, many described *forward-looking integration of the guidelines*, emphasizing that they would keep the nutritional guidance “in mind” (P033), apply it flexibly in future contexts, and adapt it to personal preferences rather than maintaining strict adherence.

**Table 2:** Subthemes and illustrative quotations related to Domain 2.

Subthemes	Illustrative Quotations
Nutritional guidance as a reference tool	*“It was helpful to have it written out, the kinds of things that are ideal to eat and not eat as reference.”* (P012)
Substitution strategies	*“I think about apples now. It was like the only fruit food or vegetable food I marked as craving. Yeah, that was a big change for me.”* (P016)
Meals as models	*“Some of the food gave me some ideas of, oh, I could make something like this myself.”* (P044)
Heightened awareness of dietary patterns	*“It kind of helped me refocus or reacquaint myself with paying attention to what I’m eating.”* (P033) *“It’s all the snacking and treats and stuff that got eliminated with this diet. So I’m focused more on snacking more healthfully, not drinking pop. So it’s more the in-between meal stuff that was the big change.”* (P031)
Forward-looking integration of the guidelines	*“There are going to be ways and restaurants and times and situations where the guidelines are going to be in my mind, they’re going to be present. So, I think that’s going to help me kind of transition, maybe not completely, but I’m going to be aware of it.”* (P033) *“I think I took away a lot of good things and I learned better that I didn’t need sugar or junk food as much as I had thought initially.”* (P010)

Note. Subthemes were derived from qualitative analysis of participant interviews. Illustrative quotes are provided as representative examples and have been lightly edited for readability while preserving participant meaning.

### 3.3. Domain 3: Palatability and Autonomy as Primary Determinants of Acceptability (Table 3)

Beyond the structural advantages of meal delivery, participants’ willingness and ability to eat the meals depended heavily on sensory appeal and perceived alignment with personal preferences. *Palatability*—including taste, seasoning, and overall enjoyment—strongly influenced willingness to eat the meals as provided. About half of the participants found dishes that they enjoyed, describing them as “tast[ing] great” (P037), “healthy” (P040), and “satisfying” (P037). However, participants’ general comments about the chosen meals suggested that the study team’s assessment of general food preferences and allergies was sometimes insufficient for appropriate selections. *Misalignment with preferences* was often noted, including some participants being provided with foods outside their norms (e.g., vegetarian/vegan meals for those who typically ate meat, a surplus of shrimp-based dishes, unfamiliar or disliked vegetables, or “unusual” spices and complex flavorings). Relatedly, participants commented on *food quality and variety*, specifically noting the absence of cold or crisp options (e.g., salads, fresh fruit) and describing meals as overly uniform (e.g., “mush”; P028, P044), especially over time. Breakfast was a particularly common pain point: about half of the participants disliked the egg-based breakfast options and avoided them altogether, while a few others noted they typically do not eat breakfast, making that portion of their delivery feel unnecessary or less useful.

**Table 3:** Subthemes and illustrative quotations related to Domain 3.

Subthemes	Illustrative Quotations
Palatability	*“The food tasted great. It filled me up…And I liked again that I had different options to choose from.”* (P037) *“Sometimes the food wasn’t very good, so it was a little bit hard to eat it.”* (P047)
Misalignment with preferences	*“The first week there wasn’t any meat at all, it was just all vegetables or vegan. I don’t eat vegan, like a vegan diet at all. And I do usually, with dinner especially, like we eat some kind of meat protein every day. So that was a lot different.”* (P026)
Food quality and variety	*“I thought the meals were good. I would have liked more variety, especially in the breakfasts. There’s a ton of repeat stuff.”* (P012) *“I didn’t feel like things were fresh because it was just all pre-cooked and packaged.”* (P040)
Mental health impact	*“I think similar to the other things, having access to healthier foods helped me feel better about what I was eating, which helped improve my mood.”* (P001) *“I think the biggest thing too is for me... I trusted that it was all healthy… I didn’t feel ashamed of anything that I was eating or guilty.”* (P044) *“Because I didn’t like much of the food, a lot of it went to waste. And that makes me feel very guilty.”* (P028)
Perceived restrictiveness	*“Quite frankly difficult. I feel as though it was really restrictive… It was like constantly kind of slightly anxiety-inducing to have to be like, “Oh wait, is this on there? Can I eat this?” And having to double and triple check things.”* (P037) *“I’m eating this restrictive food that I don’t like… I guess it would be different if I had a choice, had a selection to choose from in the meal delivery, instead of just blindly being given this food.”* (P048)
Temporal shifts in acceptability	*“The first week was fairly easy. The second week was much harder because the food kind of all tasted the same and was very mushy.”* (P028)

Note. Subthemes were derived from qualitative analysis of participant interviews. Illustrative quotes are provided as representative examples and have been lightly edited for readability while preserving participant meaning. Ellipses indicate omitted text.

These palatability challenges intersected with *perceived restrictiveness,* as limited choice led some to experience the intervention as too rigid (or, less commonly, not strict enough) despite instructions allowing supplementation and flexibility. Several participants expressed wanting more autonomy in meal selection and described that, although they had strong intentions to continue following the nutritional guidance post-study, they would implement it with more flexibility and using preferred foods (see Section 3.2). Importantly, poor palatability, misalignment, and perceived restrictiveness were not always experienced as minor inconveniences; several participants described emotional consequences that sometimes had a negative *mental health impact*, including guilt about wasted food, stress about not following the program as intended, frustration about having to prepare alternative foods, or frustration or resentment at feeling “stuck” with unwanted meals.

Finally, participants described *temporal shifts in acceptability*. Within the two-week intervention period, some found the meals to offer “a diverse set of options” (P037), whereas others described a shift from initial novelty and perceived variety to greater repetition or sensory fatigue (which diminished meal appeal for some). A shift across study enrollment was also observed: participants enrolled earlier more often described meals as good or well-seasoned and had strong favorite meals, whereas later participants more frequently characterized meals as bland, texture-repetitive, or “hard to eat” (P047), which reduced intake and increased supplementation with non-study foods. Although meal quality was not independently assessed by the study team, this temporal clustering suggests possible variability in delivery source and underscores the importance of ongoing quality control and monitored fulfillment in intervention optimization.

### 3.4. Domain 4: Social Contexts Were the Most Consistent Barrier to Adherence (Table 4)

Nearly all participants described adherence as most difficult in social contexts, highlighting several ways that eating is inherently interpersonal and situation-dependent. *Household logistics* complicated adherence when participants had to navigate separate meals within shared routines (e.g., cooking for family while eating something different) and manage schedules and shared kitchen spaces. *Social settings as temptations* were a pervasive challenge, as restaurants, celebrations, and travel increased exposure to non-concordant foods and reduced control over what was available. As a result, social events often became deviation points, with participants describing otherwise manageable adherence unraveling in these settings (e.g., “I blew it completely”; P040). Participants also emphasized *interpersonal and affective consequences*, which included sadness, stress, and feeling excluded or singled out, as well as reduced enjoyment of shared mealtime with friends. At the same time, one participant described the *use of prospective strategies* to maintain adherence in social environments, such as planning ahead by bringing a guidance-consistent dish to a potluck or discussing expectations with household members in advance, which helped reduce both practical barriers and social strain.

**Table 4:** Subthemes and illustrative quotations related to Domain 4.

Subthemes	Illustrative Quotations
Household logistics	*“It was hard to eat separately from my family… like I cooked them dinner and then had to eat my own dinner.”* (P016) *“[The biggest obstacle was] refrigerator space. I live in a house with two other people, and we had two refrigerators.”* (P048)
Social settings as temptations	*“The issue I ran into is when I had to go out of town for the weekend to celebrate with friends and I was in a social setting. And then it just fell off a cliff. It was really difficult to follow the guidelines when I was constantly busy and constantly like restaurants or bars.”* (P033) *“We had a family from out of town. It was the 4th of July. We were doing lots of family meals, and it was hard not to participate in them.”* (P043)
Interpersonal and affective consequences	*“It took out the enjoyment of being with your friends and all enjoying like a meal together… Because it ended up making them point it out that you had to eat something different than everybody else did.”* (P048) *“I was sad when other people were eating cake in front of me.”* (P003)
Use of prospective strategies	*“It was like a potluck and I planned ahead so I knew that I could make something that would adhere to the guidelines without being like, oh, why aren’t you eating what everybody else is?”* (P044)

Note. Subthemes were derived from qualitative analysis of participant interviews. Illustrative quotes are provided as representative examples and have been lightly edited for readability while preserving participant meaning. Ellipses indicate omitted text, and bracketed text indicates clarifying information added by the authors.

### 3.5. Domain 5: Withdrawal Response to Dietary Change and Temporal Adjustment (Table 5)

Participants described a time-linked adjustment process characterized by several interrelated patterns. *Early physiological disruption* was common, with the first days of the study period marked by heightened cravings and withdrawal-like symptoms (e.g., irritability, headaches, fatigue, hunger, and sleep disruption), often attributed to reduced sugar and sweetened beverage intake (mainly soda). Although a few characterized early cravings as strong—particularly among those who also experienced withdrawal—participants overall described multiple features of the intervention that helped them persist.

**Table 5:** Subthemes and illustrative quotations related to Domain 5.

Subthemes	Illustrative Quotations
Early physiological disruption	*“I did feel hungry often and I did have a few headaches…which I don’t [typically] get.”* (P047) *“I noticed there were a few times where I was extra irritable and just not really wanting to do anything. Very anxious… it would seem like I would almost need something processed or some kind of sweet or sugary food item or drink to kind of give me a little boost.”* (P043)
Study accountability	*“I was really surprised… the cravings… took like a day, maybe two at max. But… my mindset was a huge factor because I was determined to follow the guidelines… to prove to myself I could do this.”* (P044) *“I really wanted to do the best I could for the study, so I didn’t really let [cravings] impact what I ate”* (P001)
Physiological adaptation	*“The first week, actually the first three days, I was very grumpy, irritable, a little hostile. In the second week it was much easier, I noticed my mood to lighten and I felt more awake.”* (P003)
Reemergence and/or intensification of cravings	*“I was mostly successful until the end and then my cravings just became strong and overwhelming...I did wonder how much of that was knowing we were close to the end.”* (P016) *“I actually had a harder time the last two days… [I knew] I wasn’t going to have healthy three meals a day coming my way… I started to think, ‘oh, I’m free… I can eat whatever I want.’”* (P012) *“Going into the second week… it was getting better, but also difficult because I knew I was getting close to the end… once it ended, I already had a plan for how I was gonna break [the nutritional guidance].”* (P037)
Cravings in context	*“There were definitely times when I wanted to cave and it’s so difficult because we live in a world where it’s so easy to access those things…[but] as long as I knew that I was going home and I knew that I had food waiting for me…it was easy”* (P033) *“When I was more active, it was easier to not worry about food…But once I got to the point where I was hungry and I was not able to be at home or I didn’t prepare…that’s when cravings started really ratcheting up. That’s when I was like, oh, there’s Qdoba right there. Oh, there’s Chinese food right there. So, it was definitely a lot more difficult being reactive when it came to dealing with cravings.”* (P033)

Note. Subthemes were derived from qualitative analysis of participant interviews. Illustrative quotes are provided as representative examples and have been lightly edited for readability while preserving participant meaning. Ellipses indicate omitted text, and bracketed text indicates clarifying information added by the authors.

Several framed resisting cravings as a personal challenge (e.g., to see if they “could do this”; P044) or described moments of recommitment (“I was like, ‘you know what, I’m still doing this’”; P033), whereas others appreciated the *study accountability* and described their motivation to “do well” for the study or to provide “good data” as helping them persist through cravings (e.g., P003). One participant also noted the study duration as a motivator; specifically, they reported that their irritability probably would have affected their adherence to the guidance if the study was longer, but “since it was two weeks”, they were “able to pull through” (P029). A few participants also explicitly mentioned the EMA as part of this dynamic: some described the accountability of reporting dietary lapses as helpful for adhering to the nutritional guidance, whereas others reported that this repeated mood prompting was burdensome and worsened their mental state by drawing attention to how severe or persistent their symptoms felt.

Over time, many participants described *physiological adaptation*, reporting that cravings diminished after a few days or were reinterpreted as transient preferences rather than intrusive urges. However, this pattern was not uniform: a small subset described a *reemergence and/or intensification of cravings* towards the end of the study, seemingly linked to anticipation of the intervention ending and the reduction of external accountability. Participants also emphasized experiencing *cravings in particular contexts*, noting that craving intensity sometimes depended on situational factors and planning. Specifically, having compliant food waiting at home, staying busy, and packing snacks were described as protective, whereas social events, limited options, delayed eating, or inadequate planning could make cravings suddenly overwhelming and contribute to reliance on readily available ultra-processed or fast foods (see Section 3.4).

### 3.6. Domain 6: “Living Proof” of Food–Mood Coupling (Table 6)

When asked directly about mental health effects, the most commonly reported benefit was increased *energy and activation benefits*, describing increased or more stable energy that supported productivity, approach motivation, and ability to initiate daily activities. Some participants also described a link between *eating regularity and mood stability*, such that more consistent intake reduced high/low swings and “crashes” (P022) across the day. A third subtheme involved *self-evaluation shifts*, with participants describing pride, increased self-efficacy, and reduced negative self-talk and guilt about food choices; for a few, this was experienced as a sense of *mental “lightness”* and feeling more positive about their choices (P012). These lived experiences often aligned with participants’ broader perceptions of the food–mood relationship. Most participants described a *comfort-eating cycle* as a familiar pattern they recognized prior to the intervention (low mood prompting cravings for ultra-processed foods, leading to comfort eating for short-term relief, followed by sluggishness and self-criticism, and so on). A related subtheme concerned *reminder or realization* of the food-mood link for minimally processed foods: while many participants already recognized that ultra-processed ‘comfort’ foods could worsen mood over time, participating in the intervention prompted a renewed awareness that eating healthier foods could actively improve how they felt by supporting steadier energy, reducing heaviness/sluggishness, and helping interrupt the comfort-eating cycle. Intentions to continue the nutritional guidance were often grounded in these perceived benefits, particularly improvements in energy and daily functioning.

**Table 6:** Subthemes and illustrative quotations related to Domain 6.

Subthemes	Illustrative Quotations
Energy and activation benefits	*“It helped give me more energy, like more productive energy, because it made me feel better in my body, so it made me feel like I could get up and go do more stuff.”* (P022) *“I think that being more energetic allowed me to accomplish more and feel a little better.”* (P018)
Eating regularity and mood stability	*“I don’t think I had as many highs and lows because [I was] not eating the simple carbs like I didn’t get that high and then that drop.”* (P040) *“It made me aware of having more steady, not eating sugar, I think, giving more stable energy and a more stable mood throughout the day.”* (P029)
Self-evaluation shifts and mental lightness	*“I was I guess proud…like eating healthier or cleaner sometimes…Kind of like that motivation to like, okay, let’s get into a healthier habit. Let’s try and be better with this.”* (P037) *“I think it really helps to keep me just feeling lighter. I think feeling lighter, like mentally, helps a lot. And just feeling positive that I was making healthier choices for myself.”* (P012) *“I felt remarkably good…I think it’s just that I didn’t have all that negative self-talk in my head all day long about being negative about body image and food choices and stuff like that…not having that constant background chatter was amazing.”* (P031)
Comfort-eating cycle	*“If I’m just having a normal good day, I don’t feel the urge to consume things like junk food. But when I’m feeling depressed or anxious… then I want to eat bad foods. So I think initially, I enjoy it, so it makes me feel better initially. But then afterwards, I usually feel worse… you have the sugar high at first, and then you just kind of crash. You feel tired, less energy.”* (P026) *“For me, if I’m eating in a way that is sporadic or consuming more highly processed foods, I’m very sluggish and not really in the greatest mood. But when I’m actually eating consistently and having balanced meals, my mood is a lot better. I feel like I have more energy, I move more, and then I sleep better. So, I feel like they’re all kind of interconnected.”* (P034)
Reminder or realization of food-mood link	*“It’s a lot easier to see when I eat an apple… Mentally, it just doesn’t feel heavy. It feels like there’s some sort of pride… in eating fresh fruit as opposed to eating highly processed sweet foods. There’s a sense of contentment or refreshment… It’s weird that we never gravitate towards those as cravings, though.”* (P033)

Note. Subthemes were derived from qualitative analysis of participant interviews. Illustrative quotes are provided as representative examples and have been lightly edited for readability while preserving participant meaning. Ellipses indicate omitted text.

## 4. Discussion

These qualitative findings complement and extend the parent study’s quantitative results [15,16] by providing an in-depth examination of how individuals experiencing depressive symptoms experienced a short-term commercial meal delivery service designed to support adherence to minimally processed dietary guidelines. Participants’ qualitative accounts help us learn how structured meal support was integrated into daily life, understand what they perceived as helpful or challenging, and offer directions for future refinement and evaluations of meal-delivery-supported dietary interventions for depression.

### 4.1. Meal Delivery Reduced Food-Related Burden and Supported Adherence

In the quantitative findings, participants reported high adherence despite delivered meals having relatively low similarity to their typical diets (~30%) and receiving mixed evaluations of palatability [15]. The present qualitative findings help contextualize this result by showing that adherence was often supported less by preference matching or enjoyment than by reductions in the cognitive and logistical effort required to follow a minimally processed diet.

Participants consistently identified the structured scaffolding provided by the study as a central feature of the intervention. As intended, meal delivery reduced the demands associated with planning, shopping for, and preparing minimally processed meals, while the nutritional guidance provided a practical reference for making food decisions when delivered meals were unavailable, not preferred, or difficult to integrate into daily life. This reduction in food-related burden is particularly relevant in the context of depression, which is often characterized by fatigue, reduced motivation, and diminished self-regulatory capacity. Importantly, participants described the benefits of meal delivery as extending beyond feasibility. By simplifying everyday decision-making around food and preparatory tasks, the meals alleviated stress around eating and created time and “mental space” for self-care, emotion regulation, and other competing responsibilities that contributed to daily stress. These findings suggest that reduced food-related decision-making may be an important psychological pathway through which meal-based dietary interventions support individuals with depressive symptoms, although future studies are needed to evaluate this mechanism directly.

Participants also valued reliable access to minimally processed meals. Several described reassurance from having prepared meals consistently available at home and from being able to “trust” that what they were eating was healthy. For some participants, having meals provided at no cost also reduced financial burden and improved food and nutrition security during the two-week period. However, these access-related benefits are important to consider alongside the likely cost barriers of commercial meal delivery services outside a research context. Although quantitative findings indicated low intentions to continue using the specific meal delivery service after the study [15], qualitative data suggest this was often driven by concerns about cost rather than rejection of minimally processed dietary change more broadly. Given associations among low income, food insecurity, and depression [23], these findings highlight the potential importance of alternative, emerging Food is Medicine approaches that improve access to nutritious foods through medically tailored meals, groceries, produce prescriptions, or other models [24,25]. Efforts to make such approaches accessible and reimbursable, including through Medicare and Medicaid pathways, may help address access-related barriers that would otherwise limit the feasibility of meal-based dietary interventions for mental health.

### 4.2. Participant Narratives Suggest Multiple Potential Pathways Linking Dietary Change and Mental Health

Beyond convenience and adherence, participants described experiences that illuminated several potential mechanisms through which meal-delivery-supported dietary change may support mental health. Consistent with prior research emphasizing physiological mechanisms (e.g., glycemic regulation, alterations in gut microbiota composition, reduced systemic inflammation, and changes in neuroendocrine and neural signaling [2,26,27]), participants frequently described increased energy, more stable mood, and fewer “crashes” following dietary change. These accounts suggest that shifts in dietary composition may contribute to perceived improvements in affective regulation and day-to-day functioning. They also suggest that some perceived changes in energy and mood stability may occur relatively rapidly following dietary change, whereas other physiological processes likely require longer sustained dietary adherence to emerge [26].

The present findings also point to self-evaluative and behavioral mechanisms that have received less attention in prior work. Participants frequently described reductions in guilt and negative self-talk, alongside increases in self-efficacy, pride, and perceived control over eating behaviors. These cognitive shifts were often embedded within broader sociocultural contexts in which food choices are moralized and closely tied to body image, discipline, and perceived “goodness” [28,29,30,31]. In this context, engaging with the nutritional guidance was not only a behavioral change, but also an affective and identity-relevant experience. Several participants described a sense of pride or “lightness” when making choices they perceived as “healthy”, suggesting that dietary change may exert effects partly through shifts in self-evaluation and internal dialogue. Relatedly, participants described disruptions in maladaptive eating patterns, particularly comfort-eating cycles in which ultra-processed foods were used for short-term emotional relief but were followed by longer-term mood deterioration and self-criticism.

Taken together with the reductions in cognitive burden and food-related stress described above, these findings suggest that meal-based dietary interventions may affect mental health through interconnected physiological, cognitive, behavioral, and structural pathways. Future mechanism-focused research should formally examine these pathways, including whether reductions in food-related decision-making burden, cognitive load, comfort-eating cycles, and negative self-evaluation, as well as increases in self-efficacy and pride, mediate changes in depressive symptoms. Such work should also examine how these pathways interact and evolve over longer periods of dietary adherence, as some participants felt that a longer intervention would be needed to determine whether the meal delivery and corresponding dietary change had meaningful mental health benefits.

Notably, these experiences were often not interpreted by participants as the reductions in depressive symptoms observed in the quantitative data from the primary trial [15], despite being closely tied to depressive symptoms or functioning, including increased energy, reduced rumination or negative self-talk, improved emotion regulation, disrupted comfort-eating cycles, reduced stress, and greater capacity to complete daily tasks [32]. Psychoeducation may therefore be a key component of future interventions, helping individuals recognize how relatively subtle shifts in mood regulation, energy, and daily functioning may nonetheless reflect meaningful improvements in depressive symptomatology over time [33]. This interpretive support may increase the perceived value of dietary change, particularly for individuals unfamiliar with mapping experiential changes onto diagnostic constructs.

### 4.3. Intervention Fit Shaped Acceptability and Perceived Benefit, Suggesting Opportunities for Refinement

Building on these findings, participants’ narratives suggested that the acceptability and perceived benefit of the intervention depended on its fit with participants’ preferences, routines, social contexts, and changing needs over time. Across barriers, three related considerations emerged: first, the nature of the barrier itself; second, how features of the current study design may have contributed to or intensified that barrier; and third, how future interventions could be refined to preserve support while improving flexibility, autonomy, and sustainability.

#### 4.3.1. Alignment with Preferences

The most salient and commonly reported barrier was a mismatch between delivered meals and participants’ preferences, including taste, familiarity, texture, and perceived ability to adapt or supplement meals. When meals were well matched, the structured support functioned as an enabling resource that facilitated adherence and positive affective experiences. When meals were poorly matched, however, the same structure could feel restrictive, contributing to frustration, reduced engagement, guilt about not eating provided meals, or other negative emotional responses. These findings are consistent with quantitative results showing that meal tastiness was strongly associated with perceived mental health benefits [15].

Meal-preference mismatch may have been intensified by features of the current study design. Although participants completed a baseline food preference assessment, this assessment relied on a limited and somewhat idiosyncratic selection of foods, including specific plant-based meat alternatives and niche ingredients, which may have reduced its relevance to participants’ usual diets and underrepresented staple foods. Future work may benefit from more practical and individualized preference-matching methods, including open-ended assessment of participants’ typical dietary patterns, staple foods, preferred flavors and textures, and disliked foods. To reduce staff and implementation burden, GenAI-assisted intake tools may offer one approach for eliciting and organizing open-ended preference data, identifying relevant dietary constraints, and supporting participant-menu matching [34]. For example, such tools could generate a preference summary or proposed meal profile for participants to review and revise, though careful evaluation for accuracy, privacy, usability, and equity would be needed.

To further improve intervention fit, future trials may consider two complementary refinements: increasing autonomy in meal selection and more explicitly supporting intentional hybridization. When full personalization is not possible, allowing individuals to choose from a rotating menu of minimally processed options may better align meals with personal taste preferences and typical eating patterns. Intentional hybridization may further enhance flexibility and reduce feelings of restriction by encouraging participants to combine provided meals with self-selected foods—such as fruit, yogurt, snacks, fresh ingredients, culturally familiar foods, or household favorites—while remaining broadly aligned with dietary goals. This approach may also increase variety and help delay or reduce challenges that emerge over time, such as boredom, reduced novelty, and repetitiveness of meals. Although participants in the current study were encouraged to utilize this hybrid approach, several described uncertainty about how to supplement, adapt, or replace meals when necessary. Future interventions may therefore benefit from clearer hybridization guidance and stronger attention to delivery fidelity, ensuring that these options are presented consistently and with sufficient emphasis to all participants. Together, these refinements may preserve the structure and burden reduction in meal delivery while increasing flexibility and autonomy, which prior work suggests may improve intrinsic motivation and behavioral maintenance [35].

#### 4.3.2. Social and Household Considerations

Encouraging flexibility may be especially critical in social settings—such as communal meals, restaurants, holidays, or celebrations—where the majority of participants reported difficulty adhering to the guidance, as well as feelings of self-consciousness, exclusion, or reduced enjoyment. Importantly, these contexts should not be understood only as barriers to adherence. Social eating is a relational and emotionally meaningful activity that can support belonging, identity, family roles, pleasure, and connection [36,37]. Given that loneliness and perceived social isolation are strongly associated with depression [38], these findings underscore the importance of ensuring that dietary interventions do not inadvertently exacerbate social disconnection or discourage participation in meaningful shared experiences. Rather than relying solely on prospective planning strategies, such as identifying compliant restaurant options or bringing aligned foods, interventions should emphasize that certain situations, particularly social contexts, are expected parts of daily life in which flexibility and deviations are both acceptable and anticipated.

Findings also suggest that individual-level meal provision may be insufficient when participants’ eating routines are embedded within broader household food environments. Participants with partners or family members often described additional demands related to preparing separate meals or accommodating differing dietary preferences, which, in some cases, increased rather than reduced overall food-related workload. Future interventions may consider household-level adaptations, such as providing meals or grocery support for multiple household members, offering family-oriented meal options, or incorporating household meal-planning guidance. Such approaches may reduce food-related burden, promote alignment around shared meals, and support healthier household food environments. This suggestion is consistent with public perceptions of emerging Food is Medicine approaches, in which household-level support has been identified as desired, acceptable, and important for reducing burden and facilitating dietary change within families or shared living contexts [39].

More broadly, framing dietary change as adaptable rather than rigid may be critical for supporting both adherence and psychological well-being. Emphasizing overall improvements in dietary quality, rather than strict adherence to prescriptive rules, may reduce guilt, improve sustainability, and better reflect real-world eating behavior [40]. These considerations may be particularly important for individuals who are vulnerable to social withdrawal or affective sensitivity around eating behavior [41]. Although quantitative evidence from Worth et al. (2026) indicated no increase in disordered eating-related thoughts or behaviors in response to the meal delivery intervention [16], participants’ accounts add nuance by highlighting occasional experiences of perceived restriction, frustration, guilt, or social disconnection when consuming pre-selected or disliked meals, or when the intervention limited their ability to share meals with others. Importantly, these experiences were generally framed in relation to the external structure of the intervention rather than internally driven restrictive cognitions or weight/shape-related concerns. This distinction may suggest that externally scaffolded dietary programs may produce transient experiences of restriction without necessarily increasing clinically significant disordered eating pathology. However, longer observation periods are required to determine if there are long-term adverse effects of external study supports.

#### 4.3.3. Early Adaptation and Comfort Eating

The current intervention involved a rapid shift from participants’ usual diets to consistent provision of minimally processed meals, which may have intensified early adaptation experiences for some participants. A substantial proportion reported “withdrawal-like” experiences in the first few days following dietary change, including irritability, cravings, headaches, and sleep disruption [42]. These experiences suggest that the initial phase of dietary change may, for some individuals, involve short-term increases in negative affect or discomfort before stabilization occurs [43]. In clinical contexts, this underscores the potential value of front-loaded support strategies, including normalization of transient discomfort and short-term coping strategies for managing cravings, irritability, or disrupted routines [44]. Identifying individuals who may be more vulnerable to these early experiences may also be important for tailoring intervention intensity and structure.

Relatedly, participants’ narratives consistently illuminated behavioral patterns relevant to depressive symptoms; particularly the persistence of a “comfort-eating cycle,” in which negative affect and fatigue increase vulnerability to ultra-processed food consumption for short-term relief, followed by worsened mood and self-criticism. Nearly all participants described engaging in this cycle prior to the intervention, underscoring its relevance as a common lived experience in this sample. Although participants still experienced moments during the intervention when they were tempted to eat ultra-processed foods for comfort, many indicated that adherence to the nutritional guidance during these moments was supported by study accountability. This suggests that external support may be especially impactful during periods of reduced self-regulatory capacity [45]. These findings also highlight the potential value of incorporating anticipatory education that helps individuals identify emotional and contextual triggers for eating behavior and develop proactive strategies for navigating these moments once external scaffolding is reduced [46]. As previously noted, rather than emphasizing rigid control, education may benefit from a more supportive, “harm-reduction-informed” framing that prioritizes awareness, flexibility, and sustainability in real-world eating contexts [47].

#### 4.3.4. Adapting Interventions to the Individual

Together, the present findings suggest that refined meal delivery-supported dietary interventions may be a promising approach for supporting individuals experiencing depressive symptoms. However, fully prepared meal delivery is also resource-intensive, particularly with respect to cost. The heterogeneity of participants’ experiences further suggests that future interventions may be most effective when components are personalized to participants’ barriers, needs, preferences, and available resources, rather than delivered as a uniform model.

For instance, meal delivery-supported dietary interventions may be especially useful for individuals with mild to moderate depressive symptoms, those awaiting access to traditional treatments, or those in periods of “watchful waiting” in which behavioral change targets are feasible and actionable [7,48]. Participants’ short-term improvements in energy, mood stability, and productivity suggest that meal delivery may lower barriers to health-supportive routines in a manner consistent with behavioral activation principles [49]. As participants develop greater capacity to complete daily tasks, they may become better positioned to engage with more participant-involved and potentially cost-effective supports. For example, partial meal provision, meal kits, grocery support, structured meal plans with accompanying grocery lists, or other forms of hybridization may require greater effort than fully prepared meals but still preserve the key benefits of reduced decision-making burden identified in the current study and allow for greater flexibility and autonomy. In this way, fully prepared meal delivery may be most useful as an initiating support for some participants, while lower-intensity models may help sustain dietary change once participants have greater energy, symptom improvement, self-efficacy, or routine adaptation.

Just-in-time adaptive interventions may offer another strategy for tailoring the intensity and timing of dietary support [50]. Paired with therapy-based or coaching approaches, participants could learn to identify and anticipate periods when external support may be most helpful, such as episodes of low mood, reduced energy, cravings, busy schedules, or disruptions to routine. During these higher-risk periods, temporary increases in support, including prepared meals, planning tools, or external accountability, may help participants maintain dietary goals while avoiding the need for continuous full meal provision.

For individuals with more severe or complex presentations of depressive symptoms, meal-based dietary support may be most appropriate when integrated alongside existing psychosocial and pharmacological treatments, pending further research on long-term clinical outcomes and generalizability [51,52]. Ultimately, future work should examine how to tailor the timing, intensity, and type of dietary support to participant needs, including when fully prepared meals are necessary, when lower-intensity supports are sufficient, and when additional clinical support is needed.

## 5. Limitations

Several additional limitations should be considered when interpreting these findings. First, the relatively short duration of the intervention (two weeks) constrains conclusions about the sustainability of both adherence and perceived benefits. While many participants described initial adherence and early benefits, some suggested that adherence might become more difficult to maintain over time, with reports of increasing cravings and plans to “break” the diet emerging even within the final days of the intervention. These patterns highlight the need for longer-duration evaluations to better characterize trajectories of adaptation, stabilization, and potential relapse, as well as the time course over which both physiological and psychological benefits may emerge and reinforce one another. At the same time, participants’ experiences suggest that meal delivery may be best conceptualized not necessarily as a long-term standalone model, but as a short-term initiating support that can be paired with a gradual transition toward more self-managed eating.

Second, because participants experienced the nutritional guidance and meal delivery as a highly integrated intervention, it was not possible to disentangle the independent or synergistic effects of each component. Participants frequently described the meals as operationalizing the guidance, and the guidance as structuring decisions around the meals, limiting our ability to determine which elements were most influential. This has important implications for optimization and scalability, as meal delivery is resource-intensive and may not be feasible at scale for all populations or health systems [53,54]. Without clearer evidence regarding which components drive engagement and outcomes, it is difficult to determine whether similar benefits could be achieved through the lower-cost or more flexible approaches described in the previous section. Future research would benefit from designs that explicitly isolate and compare these components to inform efficient, acceptable, and scalable intervention strategies.

Several practical and implementation-related limitations also emerged that may affect scalability. Mirroring challenges reported in broader research on commercial meal delivery and meal-kit services [53,54,55,56,57], participants reported variability in food quality, palatability, and delivery experience, including issues related to packaging waste and environmental burden, challenges with packaging usability (e.g., difficulty opening meals), perceived freshness of the food, and misdelivery to neighboring addresses. Practical challenges may also vary by geographic location. Although meal delivery was available nationally in the current study, participants were required to live within approximately one hour of the study laboratory for in-person visits, generally reflecting an urban/suburban or metropolitan-adjacent population. Implementation in more remote areas may therefore differ due to transportation barriers or shipping logistics, including reliance on P.O. boxes, centralized mail delivery, or reduced home delivery reliability. These findings underscore that the real-world effectiveness of dietary interventions involving food provision may depend heavily on the consistency and reliability of external partners, which may vary over time or across settings. Potential disruptions in service, variability in food preparation, and the business sustainability of providers represent important considerations for large-scale implementation.

In addition to design features, methodological features of the qualitative design may have influenced the depth and specificity of findings. Interviews were fully structured, which limited opportunities for real-time probing or clarification of emerging themes [58]. Additionally, interviews were conducted within the context of a mental health and dietary intervention, and some participants described wanting to ‘do well’ in the study. As such, social desirability or demand characteristics may have influenced participant narratives, including the extent to which participants emphasized positive experiences or framed challenges in ways that aligned with perceived study goals [59]. While interviewers encouraged candid feedback, these contextual factors should be considered when interpreting the findings. Finally, while the use of a hybrid AI-assisted analytic approach facilitated efficient identification of initial codes, it also introduced challenges. In particular, early coding outputs tended to fragment participant narratives and obscure temporal and contextual nuances, requiring subsequent re-analysis of audio recordings to capture more complex patterns (e.g., tension between convenience and fit, shifts in meal acceptability over time, or clustering of experiences across enrollment periods). While these steps strengthened the final analysis, they are consistent with emerging perspectives on the promise and current limitations of integrating AI-assisted methods into qualitative research workflows [60].

Finally, the study sample may limit generalizability. Although efforts were made to recruit a heterogeneous sample, participants were predominantly female, non-Hispanic White, highly educated, and economically middle-class, which may constrain applicability to more diverse populations. Individuals who enroll in dietary intervention studies may also be more motivated, health-conscious, or open to behavior change than the broader population [61]. Nonetheless, participants’ reports of contextual barriers (e.g., financial constraints, household incompatibility, cultural food preferences) highlight how variation in individual circumstances may meaningfully shape how this intervention is experienced in real-world contexts and for different people [25,62]. Future research with larger and more socioeconomically and culturally diverse samples is needed to better understand how these contextual factors influence the implementation and longer-term sustainability of meal-delivery-supported interventions, particularly among populations who may benefit most from structured dietary support.

## 6. Conclusions

Dietary-quality-based interventions, which often emphasize reductions in ultra-processed foods, are increasingly being evaluated as potential treatments for depression. However, there is limited understanding of participants’ lived experiences with these approaches, particularly how individuals navigate adherence to minimally processed dietary patterns when supported through structured delivery models such as meal provision. Our findings suggest that the effectiveness of dietary-quality approaches depends not only on nutritional content, but on how dietary change is implemented and supported in real-world contexts. In particular, structured strategies such as meal delivery services may facilitate reductions in ultra-processed foods and support adherence to minimally processed diets by reducing cognitive and logistical barriers to food planning, selection, and preparation. At the same time, sustained adherence is also shaped by sensory acceptability, personalization, flexibility, and the broader cognitive, logistical, and social contexts in which eating occurs. Thus, adherence to minimally processed dietary patterns appears to be dynamically shaped by the interaction between external structure and individual-level fit, rather than either factor alone. With thoughtful implementation, meal delivery models may therefore expand scalable strategies for improving adherence to minimally processed dietary patterns, with potential downstream benefits for metabolic health, chronic disease risk, and mental health.

## Data Availability

Parent study materials are available on the OSF project page (https://osf.io/pcufr/). To protect participant confidentiality, de-identified interview transcripts will be made available upon reasonable request and contingent on approval of a data use agreement and applicable institutional requirements.

## Supplementary Materials

The following supporting information can be downloaded at: https://www.mdpi.com/article/10.3390/nu18121852/s1. Positionality Statements; Examples of foods and beverages recommended during the intervention (Table S1); Consent Evaluation of Dietary Restrictions and Preferences; Qualitative Debriefing Interview Guide; Codebook and Thematic Mapping.
