# Supplementary material for: Increasing Minimally Processed Food Intake in Depression via Commercial Meal Delivery: Qualitative Accounts of Participant Experiences
Source: Nutrients. 2026 Jun 9;18(12):1852. doi: 10.3390/nu18121852 (PMC13305699; doi:10.3390/nu18121852)
Supplement: Supplementary file 1 [file nutrients-18-01852-s001.zip › nutrients-4346635-supplementary.pdf]

Supplementary Materials for

"Increasing Minimally Processed Food Intake in Depression via Commercial Meal Delivery: Qualitative Accounts of Participant Experiences"

Table of Contents

Positionality Statements ... 3
Table S1. Examples of foods and beverages recommended during the intervention. ... 4
Consent Evaluation of Dietary Restrictions and Preferences ... 5
Qualitative Debriefing Interview Guide ... 6
Codebook ... 10
Thematic Mapping ... 16

Positionality Statements

EP is an advanced graduate student in a clinical science Ph.D. program at a large university in the midwestern United States. She is a white woman in her late 20's from the southern United States with two masters degrees. She has previous experience in qualitative research regarding psychosocial treatments for mental health disorders. She had no interaction with the participants in the study nor did she assist in any data collection for the broader trial. EP practices reflexive thematic analysis, in which her biases are acknowledged and noted during the analytic process along with the other coders.

SP is an undergraduate student in an honors program at a large university in the midwestern United States pursuing a B.S. in neuroscience with a minor in entrepreneurship. She identifies as a South Asian woman and has prior experience in qualitative research on mental health, accessibility, and well-being. Her previous work has involved supporting data collection and analysis in studies focused on mental health treatments and health equity. She did not interact directly with participants in the present study, but contributed to data coding and interpretation. SP acknowledges that her cultural and academic background may influence her understanding of the research topic and remains mindful of these factors when interpreting qualitative data.

IB is an undergraduate student at a large university in the midwestern United States. She is a multiracial woman in her early 20s from the northeastern United States. She had no interaction with the participants in the study nor did she assist in any data collection for the broader trial. She was trained by author EP in qualitative research methods and has previously worked on qualitative studies related to psychosocial treatments in mental health. IB recognizes that her social and academic background may influence her interpretations of data related to nutrition and mental health and is reflective of that throughout the coding process, using her coding team to discuss and check for potential biases.

CF is a postdoctoral research fellow at a large university in the midwestern United States. She holds a Ph.D. in social psychology, with a research focus in understanding how motivational and socioenvironmental factors interact to influence engagement and adherence to repeated health behaviors. She is a white woman in her mid-30s from the northeastern United States and acknowledges that her cultural, academic, personal background, and research interests may shape her interpretation of participants' experiences. Although she had no direct contact with participants and did not participate in data collection for the broader trial, she was responsible for data management and analysis in this study.

Supplementary Tables and Figures

Table S1. Examples of minimally processed foods and beverages recommended during the intervention and highly processed foods and beverages participants were asked to avoid as part of the nutritional guidance.

Food Category	Highly Processed Foods	Minimally Processed Foods
Recommended	Not Recommended	Recommended
Description provided to participants	Foods that have been manufactured from ingredients that do not occur in their natural state; ingredients often include added fats, added sugars, refined carbohydrates, preservatives, and artificial sweeteners and flavors	Foods that are purchased and eaten closer to their natural state.
Examples	- Foods with added sauces or syrups that contain preservatives and/or added sugar<br>- Added sugar (e.g., sugar, maple syrup, honey, corn syrup, cane juice, malt syrup/sugar, inverted sugar syrup, molasses, sucrose, fruit juice concentrates)<br>- Artificial sweeteners (e.g. aspartame, sucralose, saccharin, stevia leaf extracts, monk fruit, acesulfame potassium, xylitol, erythritol)<br>- Breaded or fried foods<br>- Cereals, breads, pastas, or crackers made with refined grains and/or including added sugar<br>- Baked goods, cookies, candy, ice cream, or other frozen desserts<br>- Combination frozen foods, such as pizza, burritos, or frozen dinners<br>- Juices, sodas, or energy drinks	- Fresh vegetables and fruits<br>- Frozen or dried vegetables and fruits packaged without added sugars<br>- Fresh or frozen meat, fish, and poultry without added sauces or breading<br>- Fresh eggs<br>- Milk, unless flavored or sweetened<br>- Beans and legumes, packaged fresh, dry, or frozen<br>- Nuts and seeds, packaged without added sugars or flavors<br>- Whole, intact grains<br>- Herbs and spices, fresh or dried<br>- Plain, unsweetened yogurt<br>- Butter or oil added when preparing foods<br>- Tea or herbal tea, packaged as tea bags or loose-leaf tea<br>- Coffee, packaged as whole coffee beans or ground

Consent Evaluation of Dietary Restrictions and Preferences

Do you have allergies to any of the following foods?

- Egg
- Sesame
- Shellfish
- Tree Nuts
- Soy
- Wheat
- None

How much would you rate the following food items on a scale of 1-6 with 1 being "like extremely" and 6 being "dislike extremely"?

Beans (all types)

Bell peppers

Dried mangoes

Chia seeds

Chicken

Herbal tea

Cilantro

Eggplant

Garbanzo beans

Mushrooms

Freeze-dried apple chips

Almonds

Quinoa

Almond flour crackers

Spelt crackers

Red meat

Dried apricots

SkinnyPop popcorn

Natural peanut butter

Rice

Roasted edamame

Squash

Turkey

Olives

Pistachios

Roasted chickpeas

Curry

Daring chicken (vegan)

Beyond Meat (vegan red meat)

Impossible Meat (vegan red meat)

Eggs

Dairy

Fish

Tree nuts

Sesame

Shellfish

Wheat

Spicy foods

Lamb

Qualitative Debriefing Interview Guide

**Starred questions were intended as quantitative ratings; however, many participants provided additional elaboration.*

Overall Response to the Intervention

For the next portion, I will be asking about how your last two weeks when we asked you to follow specific nutritional guidance.

1. *What was it like to try and follow the nutritional guidance we provided to you?*
2. *What was the best thing about being provided with nutritional guidance?*
3. *What were the biggest obstacles you encountered that made it difficult to follow the nutritional guidance?*
4. ***On a scale of 0 to 100 percent, 0 being not at all successful, and 100 being completely successful. How successful were you at following the nutritional guidance we provided?*
5. ***On a scale from 1 to 7, where 1 is made it much worse, and 7 is made it much better, what impact did the nutritional guidance we provided you have on your mental health?*
6. *Can you tell me a bit more about how you think the nutritional guidance impacted your mental health during the past two weeks?*
7. ***On a scale from 1 to 7, where 1 is not at all, and 7 is definitely, do you plan to keep trying to follow the nutritional guidance we provided now that the study is over?*
8. ***On a scale of 0 to 100 percent, with 0 being not at all, and 100 being completely the same, how similar was the nutritional guidance we provided you to your regular diet?*
9. *Can you tell me more about how similar or different you felt that the nutritional guidance we provided to you was compared to your regular diet? What was different from how you normally eat? What was the same?*

Meal Delivery-Specific Questions

10. *What was it like to participate in the meal delivery service?*
11. *What was the best thing about participating in the meal delivery service?*
12. *What were the biggest obstacles to participating in the meal delivery service?*
13. ***On a scale from 1 to 7, where 1 is made it much worse, and 7 is made it much better, what impact did participating in this meal delivery service have on your mental health?*

14. *Can you tell me a bit more about how you think having this meal delivery service impacted your mental health during the past two weeks?*
15. *What was your favorite meal delivery meal?*
16. *What was your least favorite meal delivery meal?*
17. *Were there any meals we provided that you did NOT eat? (If yes, why did you not eat these meals)?*
18. ***On a scale from 1 to 7, where 1 is tasted very bad, and 7 is tasted very good, overall how tasty were the meals we provided you?*
19. ***On a scale from 1 to 7, where 1 is not filling at all, and 7 is very filling, overall how filling were the meals we provided you?*
20. ***On a scale from 1 to 7, where 1 is not at all and and 7 is definitely, how likely would you be to continue using the meal delivery service?*
21. ***If you had to pay for the meal delivery service yourself, how much would you be willing to pay per meal?*
22. ***On a scale of 0 to 100 percent, with 0 being not at all, and 100 being completely the same. How similar was the food that we provided you through the meal delivery service to your regular diet?*
23. *Can you tell me more about how similar or different you felt that the meals we delivered to you were compared to your regular diet? What changed? What was the same?*

<u>Ultra-Processed Food Craving Questions</u>

Now I am going to ask you to rate your experiences during the past two weeks, as well as answer some follow up questions for me.

24. ***On a scale from 1 to 7, where 1 is not at all, and 7 is extremely, what were your cravings for highly processed food like during the past two weeks?*
25. *Can you tell me a bit more about what your cravings for highly processed foods were like during the past two weeks?*
26. ***On a scale from 1 to 7, where 1 is not at all, and 7 is extremely, how much were your highly processed food cravings an obstacle to following the guidelines provided by the study team?*
27. *Can you tell me more about how your experiences with highly processed food cravings played a role in how successful or unsuccessful you were at following the guidelines provided by the study team over the past two weeks?*

Negative Affect Questions

28. ***On a scale from 1 to 7, where 1 is not at all, and 7 is extremely, how likely were you to experience negative emotions like irritability or feeling down during the past two weeks?*

29. *Can you tell me a bit more about what your experiences of negative emotions were like during the past two weeks?*

30. ***On a scale from 1 to 7, where 1 is not at all, and 7 is extremely, how much would your negative emotions be an obstacle to following the guidelines provided by the study team over the past two weeks?*

31. *Can you tell me more about how you think your experiences with negative emotions played a role in how successful or unsuccessful you were at following the guidelines provided by the study team over the past two weeks?*

Withdrawal Questions

Sometimes when people cut down on consuming something they find rewarding they can experience withdrawal symptoms. Withdrawal symptoms can be physical, like headaches, fatigue, and nausea. Withdrawal can also lead to sleep problems and feeling hungrier than usual. The most common withdrawal symptoms show up in the way we feel emotionally, such as having intense cravings, increased feelings of irritability, agitation, and frustration and more moments of feeling sad or down.

32. ***On a scale from 1 to 7, where 1 is not at all, and 7 is extremely, how strong were your withdrawal symptoms like during the past two weeks?*

33. *Can you tell me a bit more about what your experiences with withdrawal symptoms were like during the past two weeks?*

34. *If you did feel like you experienced withdrawal symptoms, can you share with me what you think you were withdrawing from?*

35. ***On a scale from 1 to 7, where 1 is not at all, and 7 is extremely, how much were withdrawal symptoms an obstacle to following the guidelines provided by the study team over the past two weeks?*

36. *Can you tell me more about how you think your experiences with withdrawal symptoms played a role in how successful or unsuccessful you were at following the guidelines provided by the study team over the past two weeks?*

Unexpected Events and Reactivity

37. *Did anything unusual or unexpected happen this week that impacted your food, feelings, eating, drinking, or exercise behaviors in the past two weeks?*

38. *How did you feel this week about logging your food and drink during the Food and Feelings days with the pre- and post-reports and taking photos during the past two weeks?*

39. *How did you feel about logging your feelings when prompted during the past two weeks?*

40. *Do you feel like anything we asked you to do changed your eating behaviors, mood, or routine in the past two weeks? (If yes, how has it changed?)*

Beliefs about Food-Mood Association

Now that you are done with this part of the study, we wanted to ask you about your general thoughts about the link between what you eat and how you feel.

41. *How do you think your mood influences what or how you eat and drink?*

42. *How did you think what you eat and drink influences your mood?*

Supplementary Analysis Information

Codebook

Nutritional guidance debriefing

Subsection	Code	Definition
General experience	Convenience	Reports of overall ease, including easy delivery and reduced effort or planning.
	Mixed Food Experiences	Mixed experiences related to flavor, freshness, satiety, and satisfaction with meals.
	Logistical issues	Logistical concerns with the process, such as delivery, packaging, and community.
	Variety and Repetition	Reactions to menu variety and meal repetition over time.
Obstacles	No Obstacles	Reports of having no difficulties with the meal delivery or process.
	Packaging Difficulty	Complaints about packaging being hard to open or seal design causing frustration.
	Flavor and Texture	Reports that the meals' taste, seasoning, or texture were unappealing.
	Meal Repetition	Mentions of repetitive meals or lack of variety over time.
	Meal Preferences	Personal taste or dietary preferences making some meals unappealing.
	Delivery or Storage Issues	Logistical or practical issues related to receiving or storing the meals.
Best aspect	Convenience	Reports of overall ease, including meals being quick, ready-to-go, and reducing effort or planning.
	Food	Reports of enjoying the variety or different types of meals provided as well as general enjoyment of the meals.

	Cost Benefit	Reports of appreciating not having to pay for meals.
	Mixed Views	Reports of ambivalence or limited benefits of meals.
Compare to regular diet	Shifting away from UPFs	Shifts from processed/prepackaged foods to whole or minimally processed foods.
	Different Ingredients/Methods	Mentions of new, unfamiliar, and different ingredients not typically eaten, including how those ingredients are prepared.
	Lower Salt and Sugar	Reports of reduced sodium and sugar intake compared to normal diet.
	Flavor and Seasoning	Observations about differences in taste, seasoning, or spicing from typical home cooking.
	Variety	Comments about greater diversity in food types.
	Smaller Portion Size	Comparisons of meal size and satiety to typical eating habits.
	Unchanged Dietary Habits	Reports of overlap between the provided meals and participants' normal eating habits.
Impact on mental health	Reduced Mental Load	Describes reduced stress, mental effort, or burden due to convenience and lack of decision-making required for meals.
	Positive Mental Changes	Mentions of positive emotional or psychological effects linked to access to healthier foods and easier meal routines.
	Negative Mental Changes	Increased stress, irritation, guilt, or anxiety related to the meal delivery service.
	No Change	Participants reporting little or no noticeable effect on mental health.

Meal delivery service debriefing

Subsection	Code	Definition
General experience	Facilitated by meal access	Impact of the meal delivery service on perspectives regarding nutritional

		guidance.
	Adjustment Curve	Descriptions of the program having a change in difficulty from week 1 to week 2.
	Food Quality and Taste	Reactions to the taste, variety, or enjoyment of provided meals.
	Meal Planning	Effort required to cook, plan meals, and balance guidance with life responsibilities.
	Social Barriers	Difficulties sticking to guidance in social situations or shared environments.
	Negative emotional responses	Experience of negative emotions in relation to use of nutritional guidance.
	Positive impact	Positive outcomes or feelings of accomplishment, motivation, or physical improvement.
	Guidance Tools	Helpful lists, written instructions, and guidance materials.
Obstacles	Lack of convenience	Challenges related to lack of time, convenience, and preparation demands, both in general and regarding the provided guidelines.
	Cravings and Restrictions	Difficulties with cravings, eliminating certain foods or drinks, and dietary restrictions, sometimes in relation to emotions.
	Social barriers	Obstacles from social engagements, events, and shared environments.
	Food quality and taste	Reactions to the taste, variety, or enjoyment of provided meals.
	Cost and Resources	Financial challenges or lack of kitchen equipment.
Best aspect	Straightforward	Ease of understanding and straightforward guidance.
	Healthy Eating and Awareness	Increased health focus, eating better, and learning about foods.
	Reduced mental load	Reduction of thinking, planning of meals/snacks, and general mental load due to access to the guidance.
	Guidance Tools	Helpful lists, written instructions, and guidance materials.

	Agency	Using the guidelines in flexible or creative ways.
	Positive impact	Positive outcomes or feelings of accomplishment, motivation, or physical improvement.
Compare to reg diet	Shifting away from UPFs	Shifts from processed/prepackaged foods to whole or minimally processed foods.
	Sugar Reduction	Cutting out or reducing added sugars compared to regular diet.
	Unchanged dietary habits	Aspects of the nutritional guidance that individuals were already following in daily lives.
	Sauce Restrictions	Loss or change of sauces, condiments, and flavorings.
	Portion Size	Differences in portion sizes or satiety compared to regular diet.
	Protein Changes	Changes in protein types or frequency compared to regular diet.
	Fruit and Veggies	Reports of eating more fruits and vegetables compared to regular diet.
	Diet Routine	Differences in structure, regularity, or routine of diet.
Impact on mental health	Energy Boost	Participants reporting more energy, alertness, or productivity.
	Mood Shifts	Reports of mood changes over time, including adjustment curves and individual instances of shifting moods.
	Negative Mental Changes	Increased stress, irritation, guilt, or anxiety related to following the guidelines.
	Positive Mental Changes	Reports of accomplishment, pride, resilience, or improved self-view.
	No Change	Participants reporting little or no noticeable effect on mental health.
	Focus and Structure	Reports of structure, scheduling, or focusing effects of the guidelines.

Mental health debriefing

Subsection	Code	Definition
Cravings	Sweet Cravings	Cravings for sugary foods, desserts, or sweetened beverages.
	Savory Cravings	Cravings for salty, savory, or fast foods.
	Emotional Cravings	Cravings triggered by emotions, stress, boredom, or environment.
	Craving Shifts	Reports of cravings shifting during the study period, in either direction or in terms of intensity.
	Minimal Cravings	Reports of little to no cravings, or being able to divert thoughts easily away from fleeting cravings.
Impact of cravings on success	No Impact	Reports that cravings had little or no effect on ability to follow guidelines.
	Slip Ups	Instances where cravings led to breaking the guidelines or eating restricted foods, sometimes fueled by emotions or social situations.
	Strategy development	Using alternative foods, strategies (mental or physical), or substitutions to manage cravings.
Negative Affect	Anxiety	Reports of heightened anxiety, nervousness, or feeling on edge.
	Depression	Reports of sadness, low mood, or depression.
	Irritability	Reports of crankiness, frustration, or being short-tempered.
	External Stressors	Reports of life stressors unrelated to the study affecting emotions.
	Minimal Negative Affect	Reports of minimal negative affect or improvements in positive affect.
Impact of negative affect on success	No Effect	Reports that negative emotions did not influence ability to follow the guidelines as well as participants who did not experience negative affect during the study period.
	Minor Difficulty	Negative affect made adherence more challenging but did not prevent success.

	Emotional Eating	Negative emotions led to eating comfort or processed foods.
	Positive Effect	Reports that overcoming or reduction of negative emotions increased adherence.
	Burden	Reports of guilt, stress, or pressure tied to adhering to the guidelines/diet.
Withdrawal	No withdrawal symptoms	Reports of no symptoms aligning with the definition of withdrawal.
	Physical Symptoms	Reports of headaches, fatigue, stomach issues, hunger, or other bodily symptoms during withdrawal.
	Irritability	Reports of crankiness, frustration, or irritability linked to withdrawal.
	Adaptation	Reports of symptoms fading over time or use of strategies to adjust to the new diet.
	Withdrawing from sugar	Participant perception that they were withdrawing from sugar/sweet UPFs.
	Withdrawing from savory UPFs	Participant perception that they were withdrawing from more savory UPFs.
Impact of withdrawal on success	No Impact	Reports that withdrawal symptoms did not interfere with ability to follow the guidelines as well as participants who did not experience withdrawal during the study period.
	Obstacles	Reports that withdrawal symptoms created challenges or temptations, even if the participant was ultimately successful.

Thematic Mapping

Final domain	Initial deductive thematic category	Initial inductive theme (subtheme(s))	Brief description from initial thematizing
Domain 1: Structural scaffolding reduced preparatory burdens	Convenience & accessibility	Convenience	Participants overwhelmingly cited ease and efficiency as the strongest advantages of the meal delivery service. They described it as “super easy,” “freeing,” and “a huge relief,” emphasizing reduced mental effort, fewer food-related decisions, and time saved from grocery shopping or cooking. Many said it “simplified [their] life,” allowing them to “grab, microwave, go.” Convenience was central to both adherence and participant satisfaction. Having prepackaged, ready-to-eat meals immediately available increased dietary consistency and reduced impulsive or unplanned eating. Participants described fewer skipped meals and less snacking, since meals were “right there and easy to do.” The simplicity of heating pre-cooked meals facilitated high compliance, even among those with busy schedules or limited cooking skills. Participants described the nutritional guidance as "straightforward" and "clear" than those in the snack condition.
	Convenience & accessibility	Reduced mental load	The preplanned nature of the meals substantially lightened cognitive and emotional demands. Participants repeatedly noted that they “didn’t have to think about what to make.” This perceived mental relief contributed to both practical accessibility and emotional well-being. Having “healthy meals ready and available” seemed to enhance motivation and self-efficacy through the reduction of mental load. This psychological accessibility reinforced the practicality of the intervention beyond mere physical convenience.
	Feasibility & acceptability	Structured support (reliable access to healthy foods & structured intake)	Participants consistently reported that having meals pre-prepared and delivered made it far easier to follow nutritional guidance. The removal of barriers such as grocery shopping, recipe planning, and meal preparation increased compliance and reduced cognitive load. The service “made eating a lot quicker” and “helped [them] follow the guidelines” by reducing decision fatigue and logistical complexity. Having “ready-to-eat”, healthy meals increased regular nutritional intake for those whose depression may have previously contributed to

			inconsistent patterns.
	Feasibility & acceptability	Mismatch with preferences (lifestyle)	For a subset of participants, the prepackaged design reduced flexibility and a sense of agency in their eating routines. Taking away the creativity and autonomy of cooking or desire for other food styles (e.g., fresh versus reheated) spurred frustration in some.
	Feasibility & acceptability	Implementation friction	Most participants reported seamless delivery, describing it as "prompt" and "straightforward," though a minority encountered minor issues — such as misdelivered boxes, limited refrigerator space, or difficulty opening the packaging. These logistical challenges were exceptions rather than the rule, but they illustrate that accessibility depended not just on meal preparation ease, but on system reliability and environmental fit.
Domain 2: Nutritional guidance increased dietary awareness and skill-building	Feasibility & acceptability	Structured support (reference tool & strategy building)	Participants further noted that the clear structure of the nutritional guidelines made it easier to snack, simply checking the list and feeling confident that they were making a healthy choice. Over time, participants used the guidelines to make healthier choices that still aligned with their personal tastes and values, such as substitutions or "swaps" that might fulfill a craving or desire to eat/drink in various situations.
	Feasibility & acceptability	Nutritional awareness (modeling & heightened awareness of dietary patterns)	Participants appreciated eating "real," minimally processed foods and reducing sugar and salt intake. Several described increased awareness of food quality and improved self-perception related to healthy eating, which enhanced the perceived legitimacy and value of the intervention. The meal delivery service was framed by many as a model of high dietary quality, seeing "how you should really eat, not just how you want to eat".
	Feasibility & acceptability	Long-term sustainability (continued integration of nutritional guidelines)	Many participants went on to describe more selective continuation and adaptation of specific program components that they found especially beneficial. Rather than maintaining the full intervention with fidelity beyond the study period, participants described wanting to modify the guidelines to better fit their daily lifestyles–"keep[ing] the aspects that felt more doable" while loosening other restrictions. Several individuals also described lasting shifts in their beliefs about food and

			nutritional awareness, such as recognizing that they could reduce their reliance on sugar or “junk food” and looking forward to keeping the guidelines “in mind” within future eating contexts without feeling the need for strict adherence.
Domain 3: Palatability and autonomy as primary determinants of acceptability	Feasibility & acceptability	Meal appeal (Palatability & food quality and variety)	Sensory and preference-based satisfaction strongly influenced participants’ overall acceptance. Some enjoyed the variety and convenience, describing meals as “healthy,” “filling,” and “tasting great.” Others criticized repetitive menus, bland or unfamiliar foods, and excessive use of eggs or vegetarian options. Breakfast meals were most frequently disliked. Some participants described the flavor profile and spices used in each of the meals to be different than their usual food preferences, while others perceived the meals as more balanced than their day-to-day diet.
	Feasibility & acceptability	Mismatch with preferences (food preferences & perceived restriction)	Participants valued the simplicity but sometimes felt constrained by lack of choice in their meal deliveries. Several reported frustration or boredom from not selecting their own meals, while others desired more control over flavors, ingredients, or cultural fit.
	Feasibility & acceptability	Mixed emotional responses (mental health impact of food choices)	Disliking meals or wasting food produced guilt and irritation. Some described anxiety or mild resentment tied to perceived restriction, loss of enjoyment in eating, or lack of alignment with personal tastes.
	Feasibility & acceptability	Long-term sustainability (temporal shifts in acceptability)	While participants indicated that the intervention was feasible during the short-term study period of two weeks, they noted that it did not feel sustainable long-term due to reduced novelty and repetition of meal taste, texture, and ingredients. Some participants even noted that it became harder after the first week due to the repetition and dislike of the food.
Domain 4: Social contexts were the most consistent barrier to adherence	Convenience & accessibility	Social contexts (household logistics, social settings as temptations, interpersonal and affective consequences, & use of prospective strategies)	Several participants faced constraints related to family meal patterns, shared kitchens, or space limitations. For example, cooking separate meals for family members or managing storage in multi-person households sometimes undermined convenience. Others described social contexts as making cravings or temptations more difficult, as watching and being around people eating foods they felt restricted in delivered both interpersonal and affective consequences (feeling sad,

			left out, irritated, etc.).
Domain 5: Withdrawal response to dietary change and temporal adjustment	Feasibility & acceptability	Cravings and withdrawals (early physiological disruption, physiological adaptation, reemergence of cravings, & cravings in context)	Participants frequently described changes in hunger, cravings, and overall appetite over the two-week study period, especially towards the early end of the program. Early-on, many reported intense cravings of UPFs or reported an increase in hunger and withdrawal-like symptoms, including feeling *"extremely hungry all the time"* (P001), experiencing fatigue, irritability, headaches, and even sleep disruption. Cravings were intertwined with participant emotion states and situational contexts. Notably, many participants described a recalibration of their cravings and appetite over time. For some, cravings diminished substantially after the first few days, while others described a more qualitative shift in the way they understood and experienced their cravings across the study period. A small subset of the group described a reemergence and/or intensification of their cravings towards the end of the study.
	Feasibility & acceptability	Structured support (study accountability)	A subgroup of participants noted the study accountability (i.e., surveys, tracking diet, meeting with study team) as being a key driver and motivator for their success in following the dietary guidelines and delivered meal plan.
Domain 6: "Living proof" of food-mood coupling	Feasibility & acceptability	Living proof of the food-mood connection (comfort-eating cycle & study as reminder of food-mood link)	Participants frequently described the intervention as providing direct, experiential evidence of the relationship between food and mood, allowing them to live the connection rather than simply understand it conceptually. For some, this represented a shift in prior beliefs; others noted it as a reminder of the link between food and mood. Many participants reflected on the comfort eating cycle they found themselves getting stuck in previously.
	Feasibility & acceptability	Mixed emotional responses (energy and activation benefits, eating regulatory and mood stability, self-evaluation shifts, & mental lightness)	Positive emotions included relief, accomplishment, and motivation, particularly when participants felt healthier or more structured in their eating habits. Many participants expressed having more energy after eating as compared to their usual "sluggish" or "tired" post-meal emotions. For others, the first week brought about initial negative emotions while the second week felt easier, "lightened and more awake".
